# Tracing the evolutionary and spatial dynamics of the 2022-2023 chikungunya outbreak in Paraguay and its regional spread across the Southern Cone

**DOI:** 10.1016/j.ijregi.2026.100912

**Published:** 2026-05-08

**Authors:** Fátima Cardozo, Roque Morel, Sully Márquez, Cynthia Bernal, Alan Martínez, Adriana Valenzuela, Paúl Cárdenas, Gabriel Trueba, Alejandra Rojas, Magaly Martínez

**Affiliations:** 1 Universidad Nacional de Asunción, Instituto de Investigaciones en Ciencias de la Salud, Dr. Baez casi Dr.Villamayor, San Lorenzo, Paraguay; 2 Universidad San Francisco de Quito, Colegio de Ciencias Biológicas y Ambientales, Instituto de Microbiología, Quito, Ecuador

**Keywords:** Chikungunya virus, Paraguay, Molecular epidemiology, Genomic phylogeographic, South America

## Abstract

**Objectives:**

During 2022-2023, the chikungunya virus (CHIKV) caused a major outbreak in Paraguay. Previous genomic analyses attributed the epidemic to local circulation since early 2022, but increasing cases in neighboring countries raised questions about its origin and spread.

**Methods:**

We analyzed 517 CHIKV East/Central/South African genomes from 2014 to 2023, including 49 generated during the 2023 outbreak in Paraguay. Time-scaled phylogenetic and discrete phylogeographic analyses were used to reconstruct viral introductions, regional dissemination, and the evolutionary dynamics of the E2:V264A substitution.

**Results:**

The epidemic lineage circulating Paraguay was nested within Brazilian sequences. Three independent Brazil-to-Paraguay introductions were inferred between 2017 and 2022, but only the introduction around 2021 was associated with sustained local transmission and patterns consistent with southward spread into Argentina and Uruguay. Brazil to Paraguay was the only route with strong posterior support. The E2:V264A substitution was consistent with being present in the lineage introduced around 2021 or becoming fixed early during expansion, with no evidence of recurrent independent emergence or association with geographic dispersal.

**Conclusion:**

The 2022-2023 outbreak is consistent with the expansion of a pre-existing Brazilian lineage introduced before sustained local transmission, with phylogeographic patterns suggestive of subsequent dissemination across the Southern Cone.

## Highlights

• The 2022-2023 chikungunya outbreak in Paraguay was driven by a lineage nested within Brazilian sequences.
• A lineage introduced around 2021 was associated with sustained local transmission in Paraguay and showed patterns suggestive of southward spread.
• The E2:V264A substitution shows no evidence of recurrent independent emergence and is compatible with early or pre-existing fixation.

## Introduction

Chikungunya virus (CHIKV) is an arbovirus associated with acute and chronic arthralgia. In Paraguay, it was first confirmed in 2015, and since 2018, outbreaks have been driven by the East/Central/South African (ECSA) genotype. Four outbreaks were reported in 2015, 2016, 2018, and 2023, the latter being the largest nationally and having the highest incidence in South America [[1], [2], [3], [4]]. This study aimed to further characterize the 2023 outbreak in Paraguay and its relationship with outbreaks across the Southern Cone.

## Methods

Detailed methods are provided in Supplementary Material 1, and sequence metadata and accession numbers are listed in Supplementary Material 2.

## Results and Discussion

**Figure 1:**
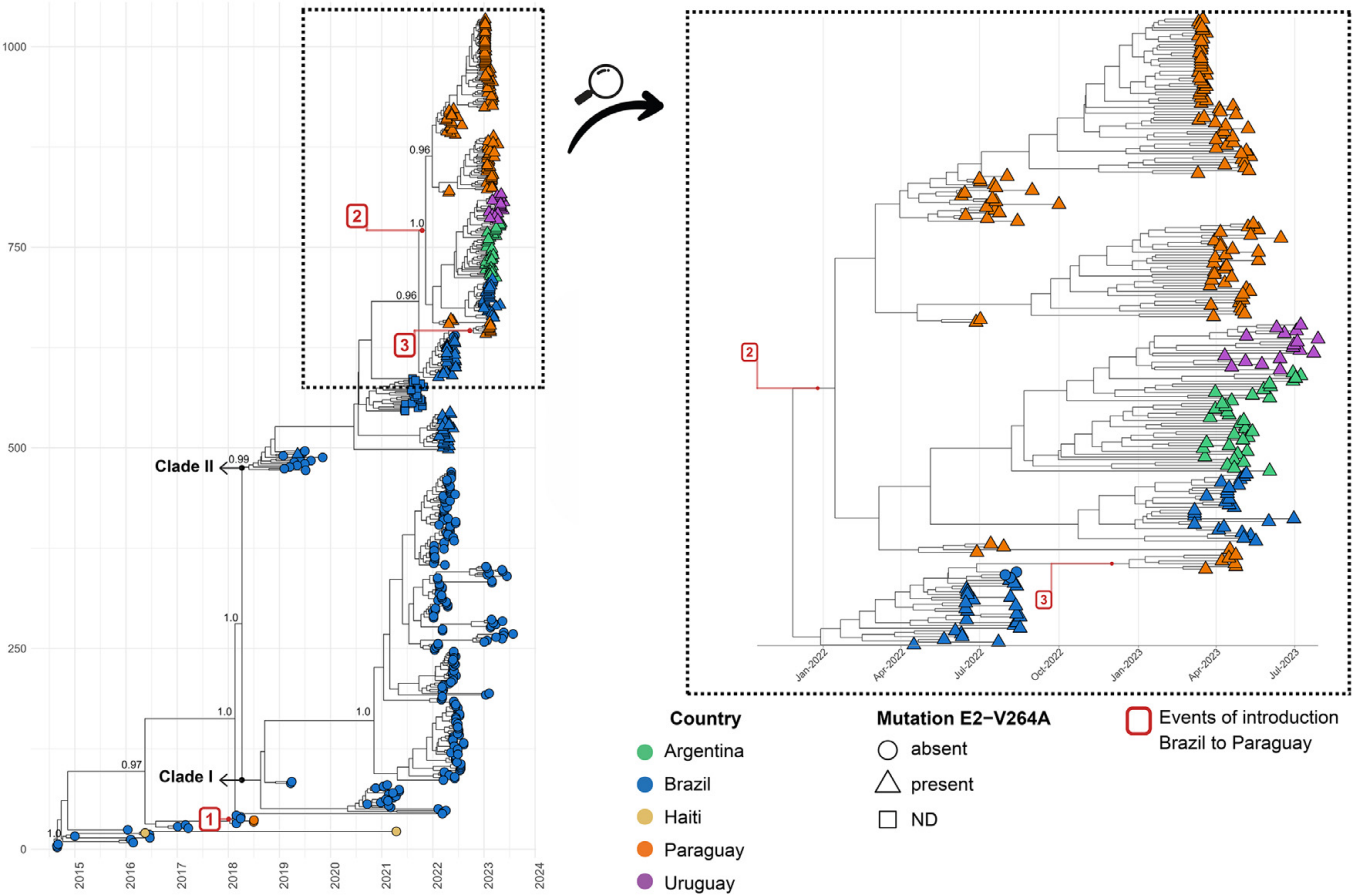
Time-scaled phylogeny of CHIKV-ECSA in the Southern Cone. MCC tree inferred from 517 near-complete CHIKV ECSA genomes sampled between 2014 and 2023. Tips are colored by country of sampling and shaped according to the E2-V264A amino acid state. Two major phylogenetic groups (clade I and clade II) are indicated, which coincide with Brazilian CHIKV-ECSA clades previously described by Xavier *et al.* [4]. Red squares denote inferred Brazil-to-Paraguay introduction events. The black dashed box indicates the region of the tree shown in detail in the zoomed panel. Posterior probability values ≥0.95 are shown for the main internal nodes. CHIV, chikungunya virus; ECSA, East/Central/South African; MCC, maximum clade credibility.

We analyzed 517 CHIKV-ECSA genomes from 2014 to 2023, including 49 generated during the 2023 Paraguay outbreak. Bayesian phylogenetic reconstruction summarized as a time-scaled maximum clade credibility tree suggests that Brazil played a central role in the early diversification of the CHIKV-ECSA lineage circulating in the Southern Cone. Consistent with previous genomic studies, the time-scaled phylogeny shows that Brazilian sequences segregate into two major clades (clade I and clade II) (Figure 1) before the emergence of the epidemic lineage responsible for the 2022-2023 outbreak in Paraguay [4]. Notably, all Paraguayan, Argentinian, and Uruguayan sequences included in this analysis clustered within a single Brazilian clade II (Figure 1). This outbreak-associated Brazilian clade II is further characterized by the presence of the E2:V264A substitution, which is absent from the Brazilian clade I. This substitution has been associated with increased viral fitness in *Aedes aegypti* when occurring with the E1:K211E mutation [5]; however, all 2022-2023 outbreak sequences analyzed here carried the E1:211T residue. In addition, *in vitro* studies have shown that variants at the E1-E2 glycoprotein interface, including the E1:211 region, can modulate virus attachment and inflammatory responses [6].

**Figure 2:**
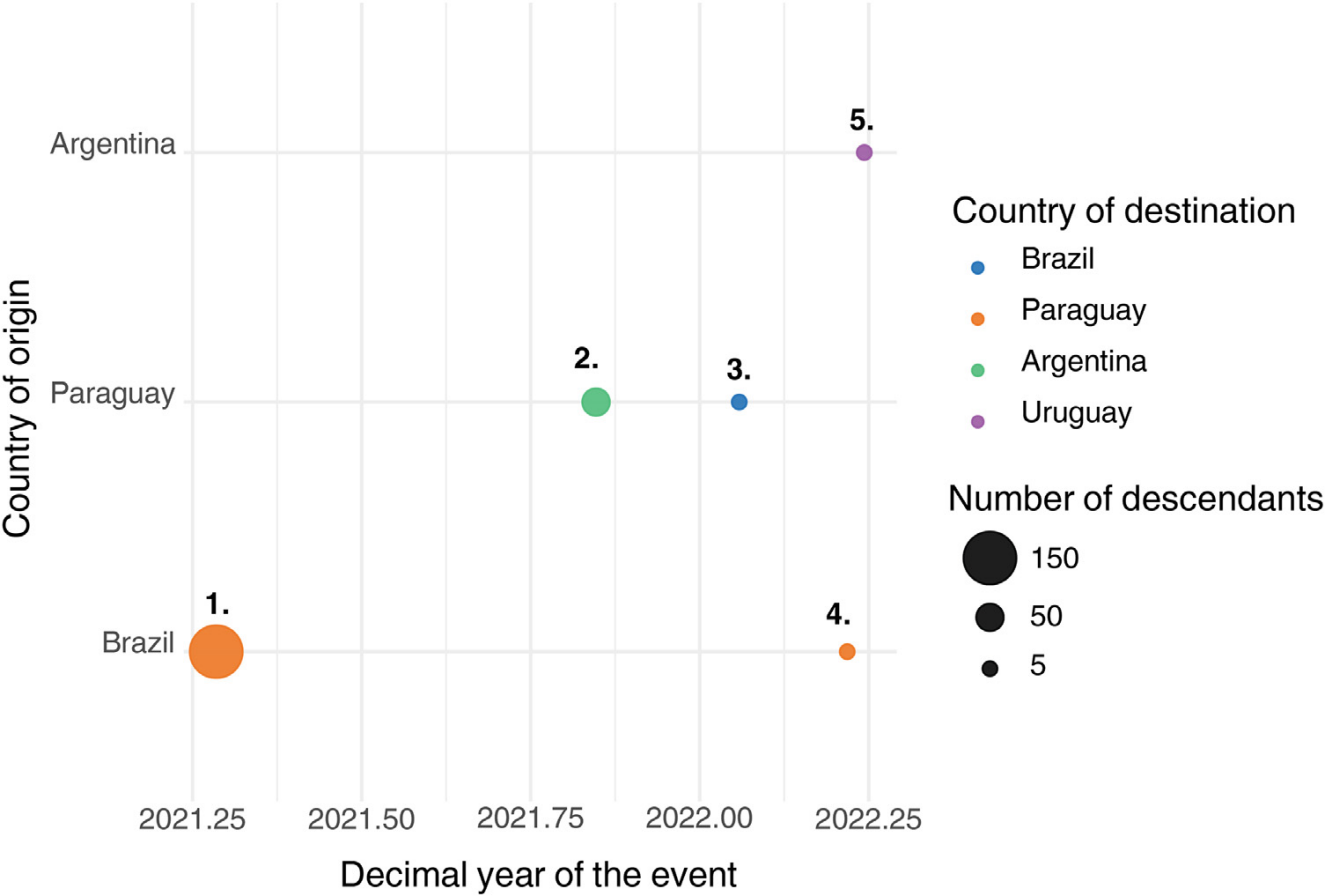
Timing and direction of inferred country-level dispersal events. Scatter plot summarizing inferred country-to-country transition events estimated from the MCC tree. The x-axis shows the decimal year of each event, the y-axis indicates the country of origin, and colors denote the country of destination. Point size is proportional to the number of descendant sequences associated with each event. Numbers label individual inferred transitions, illustrating the temporal clustering of dispersal events between 2021 and 2022. Only transitions occurring on branches with five or more descendant sequences were retained for visualization, ensuring epidemiologic relevance and reducing the impact of isolated or poorly supported events. MCC, maximum clade credibility.

Discrete phylogeographic inference identified three independent Brazil-to-Paraguay introduction events occurring between late 2017 and 2022. The earliest introduction, dated to approximately 2017 (Introduction event 1) (Figure 1), corresponds to an isolated lineage with a limited number of descendants (n = 2) (Figure 2) and no evidence of sustained onward transmission, and appears unrelated to the large outbreak observed in 2023. A second introduction around 2021 (introduction event 2) (Figure 1) was associated with the largest number of sampled descendants (n = 191) (Figure 2). Although the support for this introduction was moderate (SIMMAP support = 0.64), its large number of descendants is consistent with a major contribution to the epidemic. Mapping this major introduction onto the phylogeny suggests that it originated within the Brazilian clade II rather than from the Brazilian clade I. After this introduction, local circulation and diversification within Paraguay were followed by patterns consistent with subsequent southward spread to Argentina and Uruguay, although statistical support for these routes was moderate to low.

In contrast, the third introduction from Brazil to Paraguay in 2022 (introduction event 3) (Figure 1) resulted in a limited number of Paraguayan descendants (n = 6) (Figure 2). Further summaries of inferred geographic transitions are provided in Supplementary Material 3 (Tables S3, S4, and S6).

Bayesian stochastic search variable selection analyses of country-level dispersal routes reinforce this interpretation (Supplementary Material 3, Tables S1 and S2). Given the limited effective sample size of Markov jump counts, these analyses were interpreted primarily in terms of dominant dispersal routes rather than absolute numbers of geographic transitions. Additional summaries of geographic Markov jump analyses are provided in Supplementary Material 3 (Table S6). Among these, Brazil to Paraguay was the only transition showing high posterior inclusion probability and consistent directional support (0.99 and 0.67, respectively) (Figure 2; Supplementary Material 3, Tables S1 and S2), indicating a recurrent and epidemiologically meaningful connection between these countries. The Paraguay-to-Argentina route showed moderate directional support (directional posterior probability = 0.58; posterior inclusion probability 0.25) (Figure 2; Supplementary Material 3, Tables S1 and S2), consistent with previous genomic evidence for multiple, partially independent introductions into Argentina during recent years [2]. Other routes displayed low posterior support, suggesting sporadic or secondary movements rather than dominant transmission pathways. Directional probability estimates further indicated a preferential southward flow, consistent with patterns reported during the 2023 expansion in Argentina and Uruguay [3].

Temporal reconstruction based on node-associated dates places the mean timing of Brazil-to-Paraguay transitions around 2021, with individual events spanning several years. Subsequent movements from Paraguay into Argentina (∼2022) and later into Uruguay (∼2022-2023) are consistent with a stepwise regional propagation pattern rather than rapid, simultaneous dissemination across the Southern Cone (Figure 2). The cluster of Brazilian sequences (2023) within clade II may reflect cross-border transmission from Paraguay to Brazil, although this inference should be interpreted cautiously given the limited support for alternative dispersal routes. This pattern aligns with epidemiologic observations and is consistent with sustained local transmission in Paraguay following introduction rather than supporting an independent epidemic lineage.

The analysis of amino acid state evolution of the E2:V264A substitution indicated that posterior transition rates between absent and present amino acid states were similar, with wide and overlapping 95% highest posterior density intervals, indicating no clear directional trend (Supplementary Material 3, Table S5). Markov jump analyses further indicated a limited number of state transitions across the phylogeny, with one to two gain events and rare inferred losses (Supplementary Material 3, Table S7). These results are compatible with the E2:V264A substitution already being present in the lineage introduced from Brazil to Paraguay around 2021 or becoming fixed early during its expansion; however, the available data do not allow us to distinguish between these scenarios, and the apparent near fixation limits the assessment of its relationship with geographical dispersal.

Previous genomic analyses based primarily on Paraguayan sequences interpreted the 2022-2023 epidemic as resulting from prolonged local transmission after an introduction in 2022 and found limited evidence for cross-border transmission from Brazil [1]. By incorporating a broader regional data set and explicit phylogeographic reconstruction, our analyses add resolution to this scenario by showing that the epidemic lineage is nested within Brazilian sequences and is consistent with a dominant Brazil-to-Paraguay introduction event occurring before 2022 and subsequent sustained local transmission.

CHIKV population dynamics among neighboring countries may be shaped by cross-border movement, shared environments, and vector populations. In addition, introductions of CHIKV from Brazil into Paraguay have been previously documented [7,8].

## Conclusion

Our phylogeographic analyses indicate that CHIKV-ECSA circulation in the Southern Cone involved multiple introductions from Brazil into Paraguay between 2017 and 2022, with heterogeneous persistence across events. One introduction around 2021, involving a lineage that had circulated in Brazil before the 2022-2023 outbreak, was associated with sustained transmission in Paraguay, and this introduction is compatible with a potential role in subsequent cross-border transmission into Argentina and Uruguay.

The E2:V264A substitution, which characterizes the epidemic lineage, appears to be nearly fixed within this lineage, with no evidence of recurrent independent emergence or a detectable effect on geographic dispersal, although the timing of fixation could not be resolved from the available data. Nevertheless, its presence within the outbreak-associated lineage provides relevant evolutionary context, particularly given its previously reported functional effects.

Together, these findings are consistent with the 2022-2023 outbreak resulting from the expansion of a pre-existing Brazilian lineage within a broader epidemiologic context in which viral lineage, ecological conditions, and human mobility likely interacted to enable sustained transmission and regional spread.

## Declaration of competing interest

The authors have no competing interests to declare.

## Appendix Supplementary materials

Image, application 1

Image, application 2

Image, application 3
