# Supplementary material for: Tracing the evolutionary and spatial dynamics of the 2022-2023 chikungunya outbreak in Paraguay and its regional spread across the Southern Cone
Source: IJID Reg. 2026 May 8;19:100912. doi: 10.1016/j.ijregi.2026.100912 (PMC13273689; doi:10.1016/j.ijregi.2026.100912)
Supplement: Supplementary file 1 [file mmc1.docx]

**Tracing the evolutionary and spatial dynamics of the 2022–2023 chikungunya outbreak in Paraguay and its regional spread across the Southern Cone**

**Supplementary Material 1 - Methods**

**Sample processing protocols, Analysis, and Phylogenetics of CHIKV Sequences**

A total of 49 chikungunya virus (CHIKV) positive samples with a cycle threshold (Ct) value below 26, as determined by qRT-PCR (1), were selected for sequencing. These samples were collected as part of the project “Detection and characterization of flavivirus and alphavirus infections in suspected acute cases of arboviruses or neurological diseases, Central Department, Paraguay”. The study protocol was reviewed and approved by the Scientific and Ethics Committee of the Instituto de Investigaciones en Ciencias de la Salud, Universidad Nacional de Asunción (IICS-UNA, IRB00011984).

**Viral RNA Extraction and cDNA Synthesis**

Viral RNA was primarily extracted using the RNA/DNA Purification Kit (Magnetic Beads) – DaAnGene on the automated Smart32 Nucleic Acid Extraction instrument (DaAnGene, China), following the manufacturer's instructions. Additionally, RNA extraction was performed using the RNA preservation and purification protocol described by Hernández et al. (2). cDNA synthesis was carried out using the LunaScript RT SuperMix Kit (New England Biolabs). Tiling genome amplification was performed using Q5 High-Fidelity Hot-Start DNA Polymerase (New England Biolabs), following a previously described CHIKV-specific primer scheme for whole-genome amplification (3).

**Library Preparation and Sequencing**

DNA libraries were prepared using the EXP-NBD196 native barcode kit and the Ligation Sequencing Kit SQK-LSK109 (Oxford Nanopore Technologies plc, ONT), adapting the ARTIC SARS-CoV-2 low-cost approach (3). The library was loaded onto an R9.4 flow cell (FLO-MIN106, ONT) and sequenced using the MinION Mk1B device. Basecalling (model dna_r9.4.1_450bps_sup) and barcoding were performed using Guppy suite (Oxford Nanopore Technologies plc.), version 6.5.7+ca6d6af.

**Genome Assembly and Consensus Generation**

Reads were aligned to a reference genome using minimap2, and alignment files were processed and sorted with SAMtools (4,5). Consensus sequences were generated with iVar from mpileup data, considering bases with a minimum Phred quality score of 20 and a minimum allele frequency of 50%, while positions not meeting these criteria were masked as Ns (6). A detailed Nextflow (7) bioinformatics pipeline, including tool versions, is publicly available for inspection and cloning at <https://github.com/Seq-IICS/get_consensus>.

**Phylogenetic and Evolutionary Analyses**

The 49 CHIKV genomes generated were analyzed together with 468 publicly available CHIKV East/Central/South African (ECSA) genomes (total n=517; 2014-2023). Metadata for newly generated sequences and GenBank accession numbers for all sequences included in the analysis are provided in Supplementary Material 2.

Consensus sequences were aligned using MAFFT v7.453 and manually curated in AliView v1.28 (8,9). The alignment was subsequently filtered using TrimAl, retaining only sites represented in at least 50% of the sequences and sequences covering ≥85% of those retained positions (10). Time-scaled phylogenetic reconstruction was performed using a Bayesian Markov chain Monte Carlo (MCMC) framework implemented in Bayesian Evolutionary Analysis Sampling Trees (BEAST) v1.10.4 (11) to infer the evolutionary relationships and temporal structure of CHIKV-ECSA genomes circulating in the Southern Cone. Analyses were conducted under a TN93 (12) nucleotide substitution model with gamma-distributed rate heterogeneity across sites, an uncorrelated lognormal relaxed molecular clock, and a Gaussian Markov random field (GMRF) Bayesian Skygrid (13) coalescent prior to model changes in effective population size through time. Three independent MCMC runs were performed for phylogenetic reconstruction in BEAST. The resulting log and tree files were combined using LogCombiner after removal of 25% burn-in (14). A maximum clade credibility (MCC) tree was summarized using TreeAnnotator (Figure 1).

Country-level dispersal events were identified on the MCC tree by reconstructing ancestral geographic states and locating branches where state transitions occurred. Event timing was estimated through interpolation of branch dates, and the number of descendant sequences associated with each event was determined from the MCC topology using the ggtree package in R. These inferred dispersal events are summarized in Figure 2 and reported in Supplementary Material 3 (Table S3). The robustness of inferred country-level dispersal events highlighted in the phylogeny was further evaluated using stochastic character mapping (SIMMAP) with 100 replicate simulations conditioned on the fixed MCC tree. Approximate conditional support for individual Brazil-to-Paraguay introduction events is reported in Supplementary Material 3 (Table S4).

To investigate dispersal-route support and mutation dynamics, Bayesian discrete-trait analyses were conducted in which geographic location (country group) and the E2:V264A amino acid state were modeled as discrete traits under an asymmetric continuous-time Markov chain model (CTMC) implemented in BEAST. Bayesian stochastic search variable selection (BSSVS) was used to identify supported transition routes. Posterior estimates of country-level transition rates, inclusion probabilities, and directional support are reported in Supplementary Material 3 (Tables S1 and S2), while posterior estimates of E2:V264A amino acid state transition rates are reported in Supplementary Material 3 (Tables S5). Posterior summaries for these analyses were derived from BEAST log files, with an additional 10% of samples discarded during post-processing prior to calculating summary statistics.

Markov jump counts for country-level transitions and E2:V264A mutation state changes were estimated in independent BEAST analyses run specifically for jump count inference and not used for phylogenetic reconstruction. Posterior summaries of Markov jump counts were calculated after discarding 10% burn-in during post-processing and are reported in Supplementary Material 3 (Tables S6 and S7). Sequences with undetermined E2:V264A status (ND) were treated as missing data for biological interpretation.

Data visualization was performed using the ggtree and ggplot2 packages in RStudio 2023.09.0 (15,16). These tools were used to visualize time-scaled phylogenies, annotate ancestral state reconstructions, and display inferred country-level dispersal events, including their estimated timing and associated numbers of descendant sequences.

**Amino Acid Sequence Analysis**

Amino acid sequences from the 517 genomes included in the time-scaled phylogenetic tree were analyzed. E1 and E2 regions were aligned using MAFFT v7.453 and visually inspected against the chikungunya virus ECSA lineage reference sequence NC_004162.2 (S27 African prototype). The presence or absence of the E2:V264A substitution was determined directly from the amino acid alignment by inspecting the corresponding residue position relative to the reference sequence and encoded as a discrete character for downstream analyses (8).

**References**

1. Waggoner JJ, Gresh L, Mohamed-Hadley A, Ballesteros G, Vargas Davila MJ, Tellez Y, et al. Single-Reaction Multiplex Reverse Transcription PCR for Detection of Zika, Chikungunya, and Dengue Viruses. Emerg Infect Dis. 2016;22(7):1295.

2. Hernandez S, Cardozo F, Myers DR, Rojas A, Waggoner JJ. Simple and Economical Extraction of Viral RNA and Storage at Ambient Temperature. Microbiol Spectr. 2022;10(3).

3. Quick J. nCoV-2019 sequencing protocol v1 [Internet]. 2020. Available from: https://www.protocols.io/view/ncov-2019-sequencing-protocol-bp2l6n26rgqe/v1

4. Li H. New strategies to improve minimap2 alignment accuracy. Bioinformatics. 2021;37(23):4572–4.

5. Danecek P, Bonfield JK, Liddle J, Marshall J, Ohan V, Pollard MO, et al. Twelve years of SAMtools and BCFtools. Gigascience. 2021;10(2):1–4.

6. Grubaugh ND, Gangavarapu K, Quick J, Matteson NL, De Jesus JG, Main BJ, et al. An amplicon-based sequencing framework for accurately measuring intrahost virus diversity using PrimalSeq and iVar. Genome Biol. 2019;20(1):1–19.

7. DI Tommaso P, Chatzou M, Floden EW, Barja PP, Palumbo E, Notredame C. Nextflow enables reproducible computational workflows. Nat Biotechnol 2017;35(4):316–9.

8. Katoh K, Rozewicki J, Yamada KD. MAFFT online service: multiple sequence alignment, interactive sequence choice and visualization. Brief Bioinform. 2019;20(4):1160–6.

9. Larsson A. AliView: a fast and lightweight alignment viewer and editor for large datasets. Bioinformatics. 2014;30(22):3276–8.

10. Capella-Gutiérrez S, Silla-Martínez JM, Gabaldón T. trimAl: a tool for automated alignment trimming in large-scale phylogenetic analyses. Bioinformatics. 2009;25(15):1972–3.

11. Suchard MA, Lemey P, Baele G, Ayres DL, Drummond AJ, Rambaut A. Bayesian phylogenetic and phylodynamic data integration using BEAST 1.10. Virus Evol. 2018;4(1).

12. Tamura K, Nei M. Estimation of the number of nucleotide substitutions in the control region of mitochondrial DNA in humans and chimpanzees. Mol Biol Evol. 1993;10(3):512–26.

13. Hill V, Baele G. Bayesian Estimation of Past Population Dynamics in BEAST 1.10 Using the Skygrid Coalescent Model. Mol Biol Evol. 2019;36(11):2620–8.

14. Rambaut A, Drummond AJ, Xie D, Baele G, Suchard MA. Posterior Summarization in Bayesian Phylogenetics Using Tracer 1.7. Syst Biol. 2018;67(5):901.

15. Yu G, Smith DK, Zhu H, Guan Y, Lam TTY. ggtree: an r package for visualization and annotation of phylogenetic trees with their covariates and other associated data. Methods Ecol Evol. 2017;8(1):28–36.

16. Wickham H. Ggplot2 : elegant graphics for data analysis. Springer; 2009. 212 p.
