# Supplementary material for: Tracing the evolutionary and spatial dynamics of the 2022-2023 chikungunya outbreak in Paraguay and its regional spread across the Southern Cone
Source: IJID Reg. 2026 May 8;19:100912. doi: 10.1016/j.ijregi.2026.100912 (PMC13273689; doi:10.1016/j.ijregi.2026.100912)
Supplement: Supplementary file 3 [file mmc3.docx]

**Tracing the evolutionary and spatial dynamics of the 2022–2023 chikungunya outbreak in Paraguay and its regional spread across the Southern Cone**

***Supplementary Material 3 - Additional Results***

This Supplementary Material provides detailed posterior summaries and complementary results supporting the phylogeographic and mutational inferences described in the main text, including country-level dispersal rates, directionality, timing of inferred transitions, and mutation state dynamics.

**Table S1. Posterior mean transition rates and inclusion probabilities for country-level dispersal routes**

Mean rate refers to the posterior mean of the relative transition rate between discrete locations. Uncertainty is summarized using 95% highest posterior density (HPD) intervals. Inclusion probability represents the proportion of Markov chain Monte Carlo (MCMC) samples in which a given transition rate was non-zero.

| Origin | Destination | Mean rate | 2.5% HPD | 97.5% HPD | Inclusion probability |
| --- | --- | --- | --- | --- | --- |
| Brazil | Paraguay | 1.11 | 0.16 | 3.20 | 0.99 |
| Paraguay | Argentina | 0.89 | 0.02 | 3.42 | 0.25 |
| Paraguay | Brazil | 0.92 | 0.03 | 3.38 | 0.39 |
| Argentina | Uruguay | 0.92 | 0.02 | 3.46 | 0.24 |
| Brazil | Haiti | 0.80 | 0.07 | 2.53 | 0.97 |

Only transitions discussed in the manuscript are shown.

**Table S2. Directional posterior probabilities of country-level dispersal**

Directional posterior probability represents the proportion of posterior samples in which the estimated transition rate from origin to destination exceeded the reverse direction. This metric reflects relative directional support and should not be interpreted as formal statistical significance or Bayesian stochastic search variable selection (BSSVS) inclusion probability.

| Origin | Destination | Directional probability |
| --- | --- | --- |
| Brazil | Paraguay | 0.67 |
| Paraguay | Argentina | 0.58 |
| Uruguay | Argentina | 0.63 |
| Paraguay | Brazil | 0.42 |
| Argentina | Brazil | 0.48 |

**Table S3. Timing of inferred country-level dispersal events on the MCC tree.**

Mean timing of country-to-country dispersal events inferred on the maximum clade credibility (MCC) tree. Mean year represents the average estimated timing of transitions per route, and n events correspond to the number of distinct transitions observed on the MCC topology.

| Origin | Destination | Mean year | n events |
| --- | --- | --- | --- |
| Brazil | Paraguay | 2020.0 | 3 |
| Paraguay | Argentina | 2022.0 | 1 |
| Paraguay | Brazil | 2022.0 | 1 |
| Argentina | Uruguay | 2022.0 | 1 |

Event timing and event counts are inferred on the MCC tree and are conditional on a fixed topology. Corresponding posterior mean transition rates and inclusion probabilities for each route are reported in Supplementary Table S1.

**Table S4. Stochastic character mapping SIMMAP-based support for independent Brazil–Paraguay introduction events.**

SIMMAP-based support represents the proportion of simulations in which a Brazil-to-Paraguay transition was inferred on the corresponding branch.

| Event ID | Estimated year | SIMMAP-based support |
| --- | --- | --- |
| 1 | 2017.9 | 1.00 |
| 2 | 2021.3 | 0.64 |
| 3 | 2022.2 | 1.00 |

The corresponding introduction events are indicated on the time-scaled phylogeny shown in Figure 1 of the main manuscript. SIMMAP-based support reflects uncertainty in ancestral state reconstruction conditional on the MCC tree and does not integrate over uncertainty in tree topology or model parameters. Values of 1.00 indicate that the transition was recovered in all simulations given the fixed MCC topology, rather than absolute certainty.

**Table S5. Posterior estimates of E2V264A amino acid state transition rates.**

| Transition | Mean rate | 2.5% HPD | 97.5% HPD |
| --- | --- | --- | --- |
| Absent → Present | 1.01 | 0.058 | 3.35 |
| Present → Absent | 0.95 | 0.084 | 3.01 |

ND states were treated as missing for biological interpretation; therefore, absent/present transitions are shown.

**Table S6. Markov jump counts for country dispersal routes**

| Origin | Destination | Mean number of jumps | 2.5% HPD | 97.5% HPD |
| --- | --- | --- | --- | --- |
| Brazil | Paraguay | 2.1 | 1 | 3 |
| Paraguay | Argentina | 1.0 | 0 | 2 |
| Paraguay | Brazil | 1 | 0 | 2 |
| Argentina | Uruguay | 1 | 0 | 2 |

Markov jump counts summarize dominant country-level transition patterns and should be interpreted as relative measures of dispersal intensity rather than exact numbers of historical events.

**Table S7. Markov jump counts for E2V264A mutation states changes**

| Transition | Mean number of jumps | 2.5% HPD | 97.5% HPD |
| --- | --- | --- | --- |
| Absent → Present | 2.1 | 1 | 3 |
| Present → Absent | 1.2 | 1 | 3 |

ND states were treated as missing for biological interpretation. Markov jump counts summarize dominant mutation state transition patterns and should be interpreted in conjunction with rate estimates (Table S5) and the phylogenetic reconstruction.
